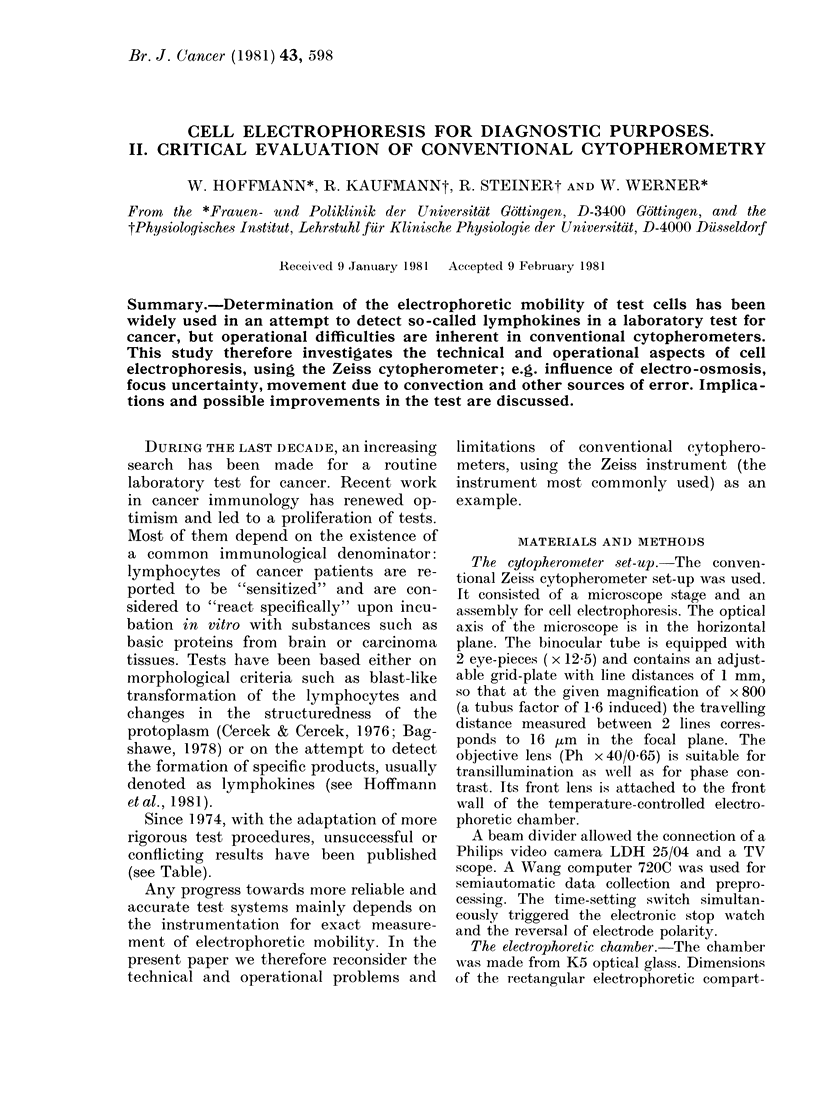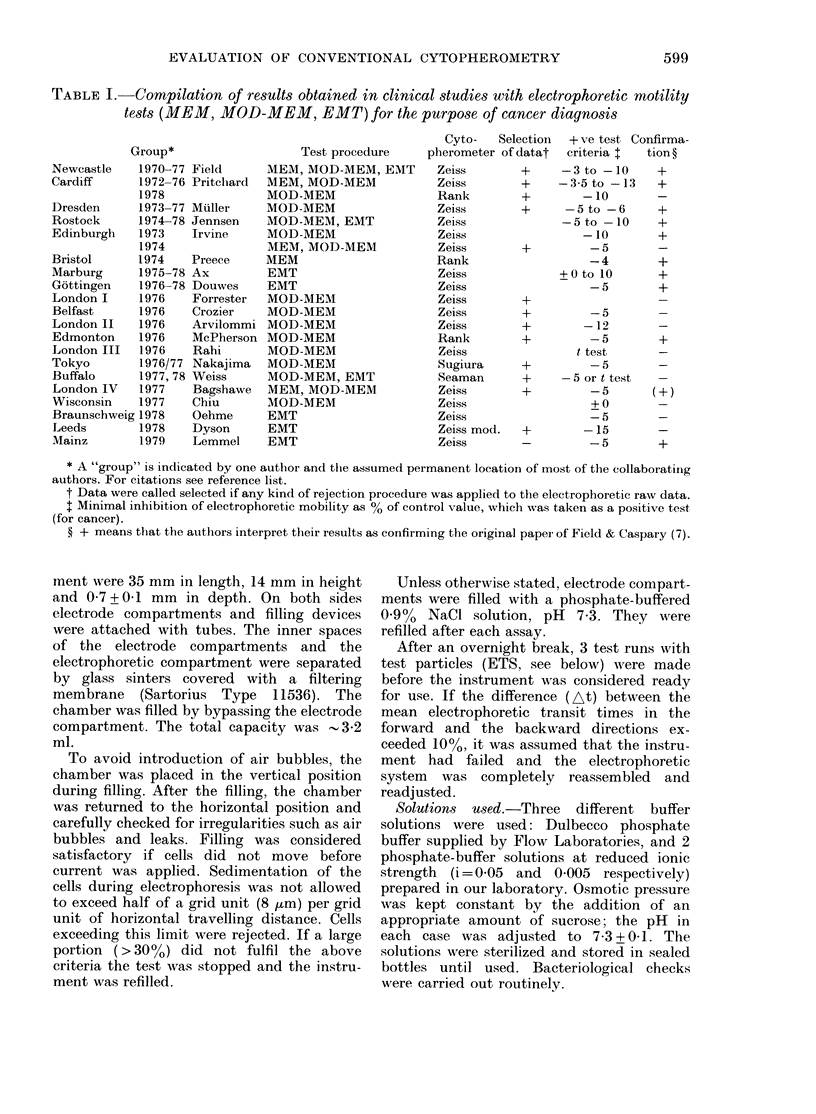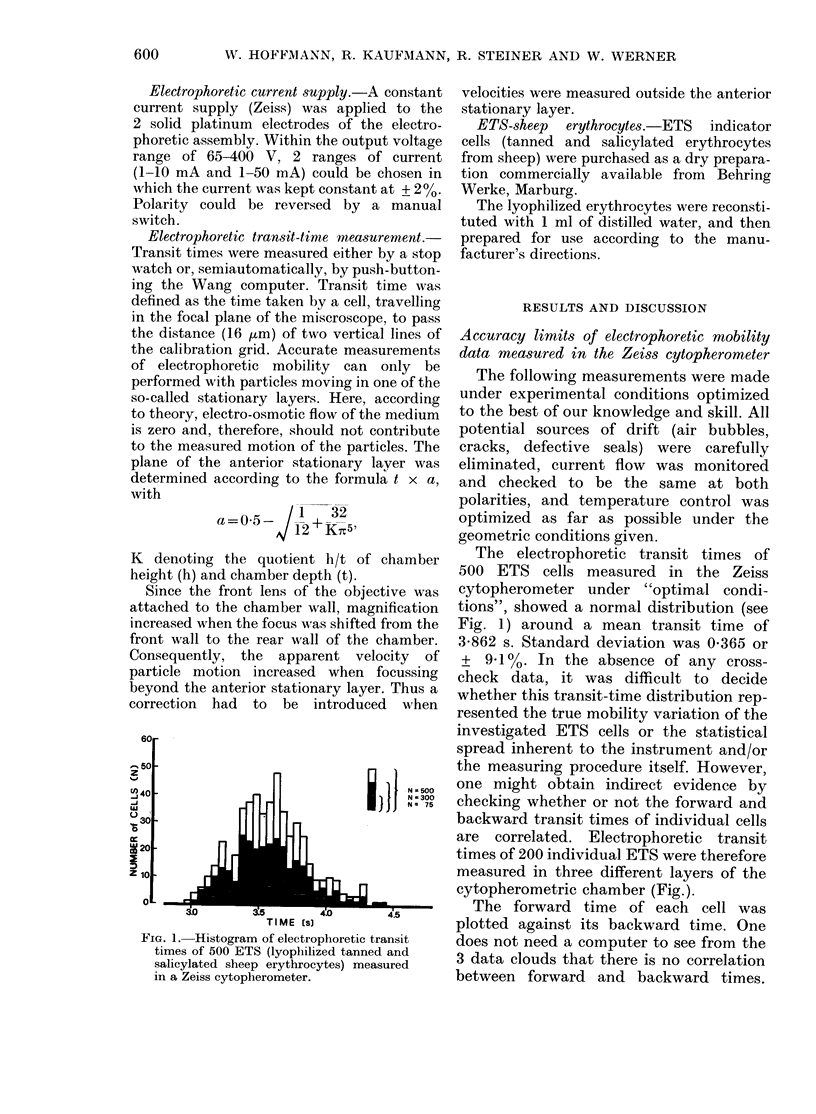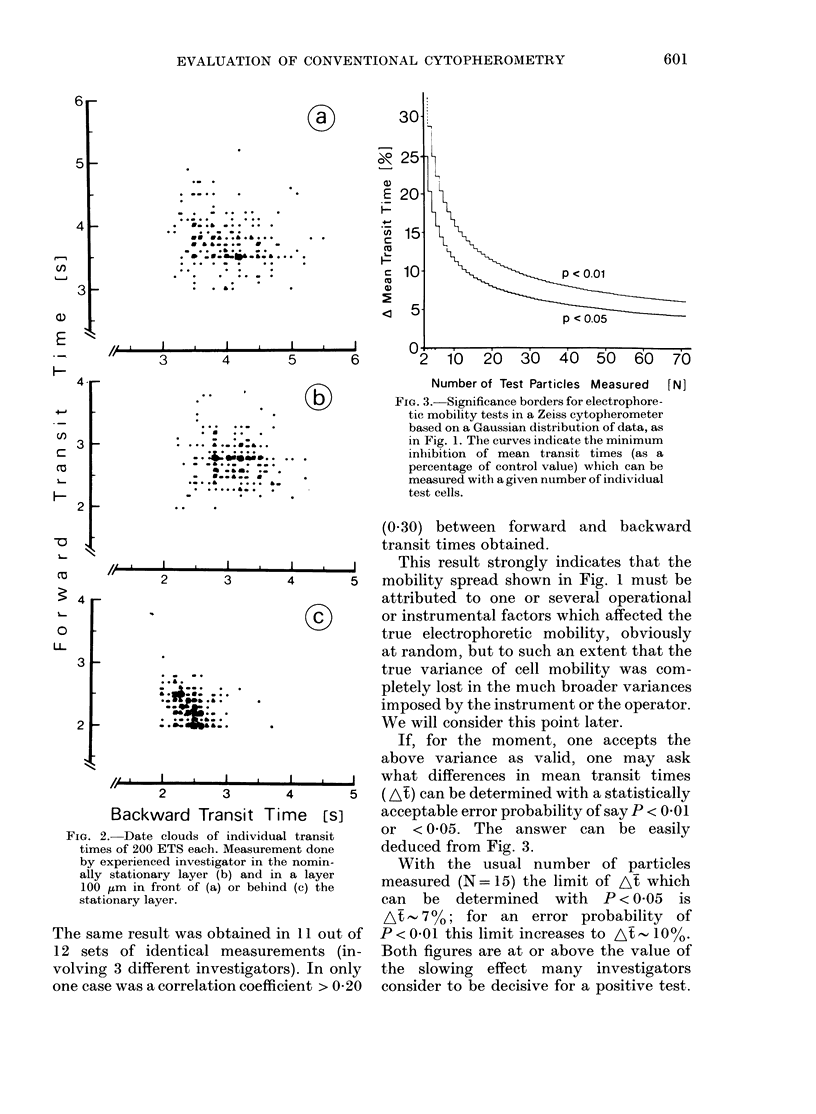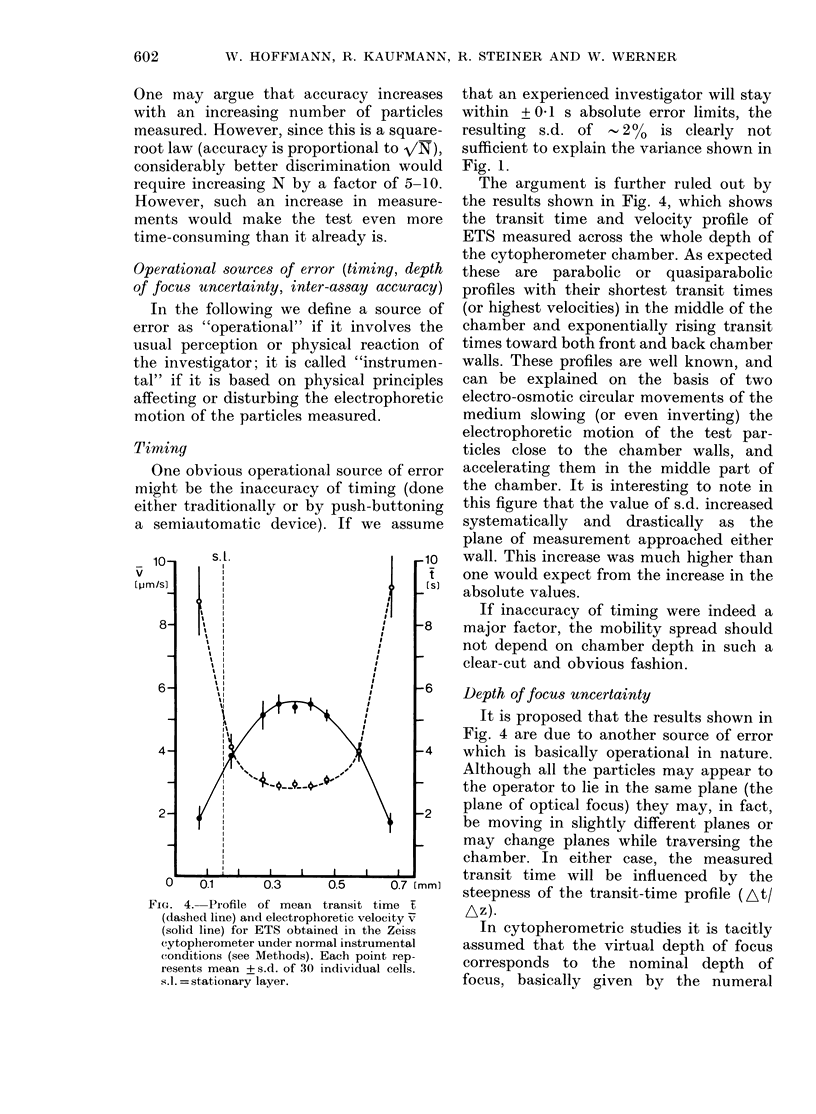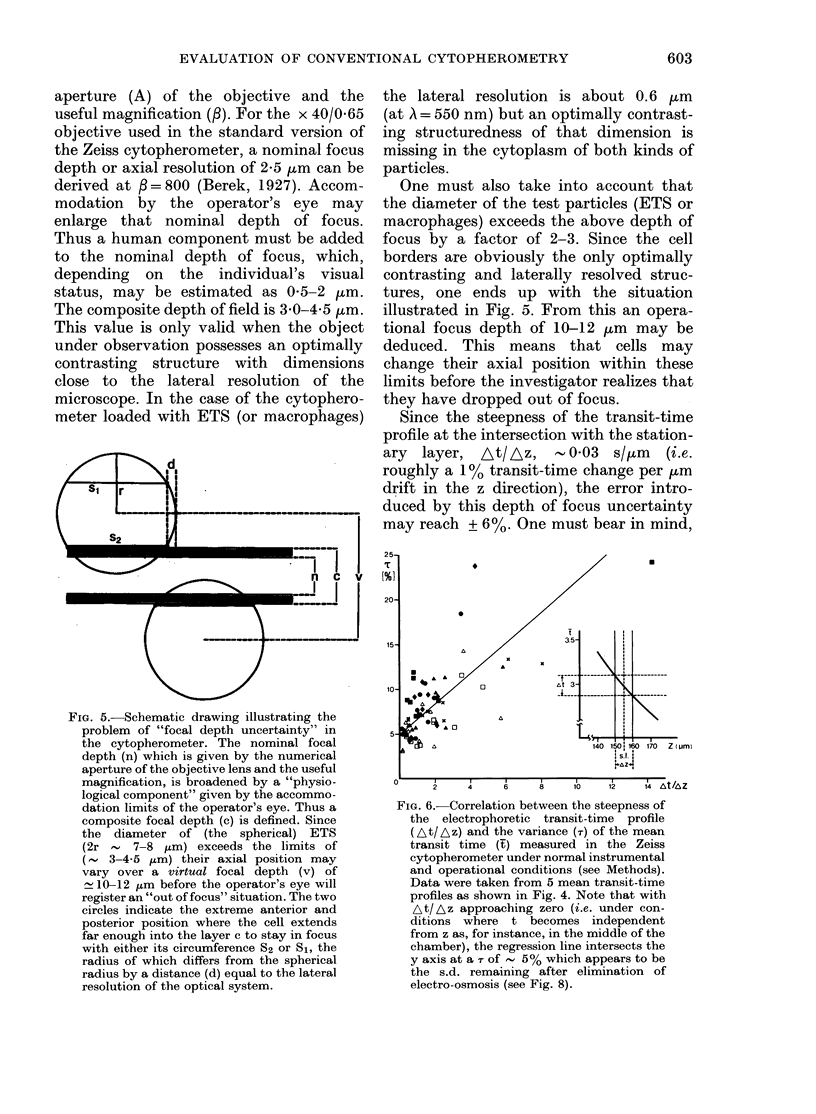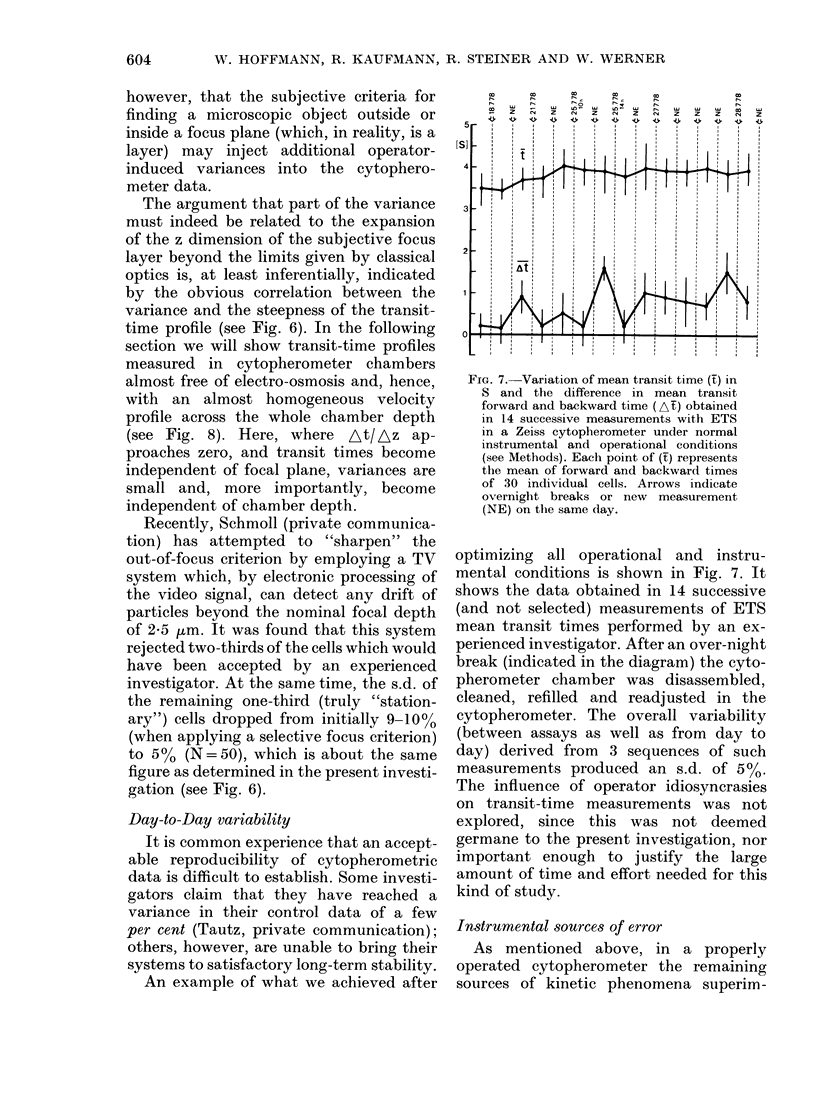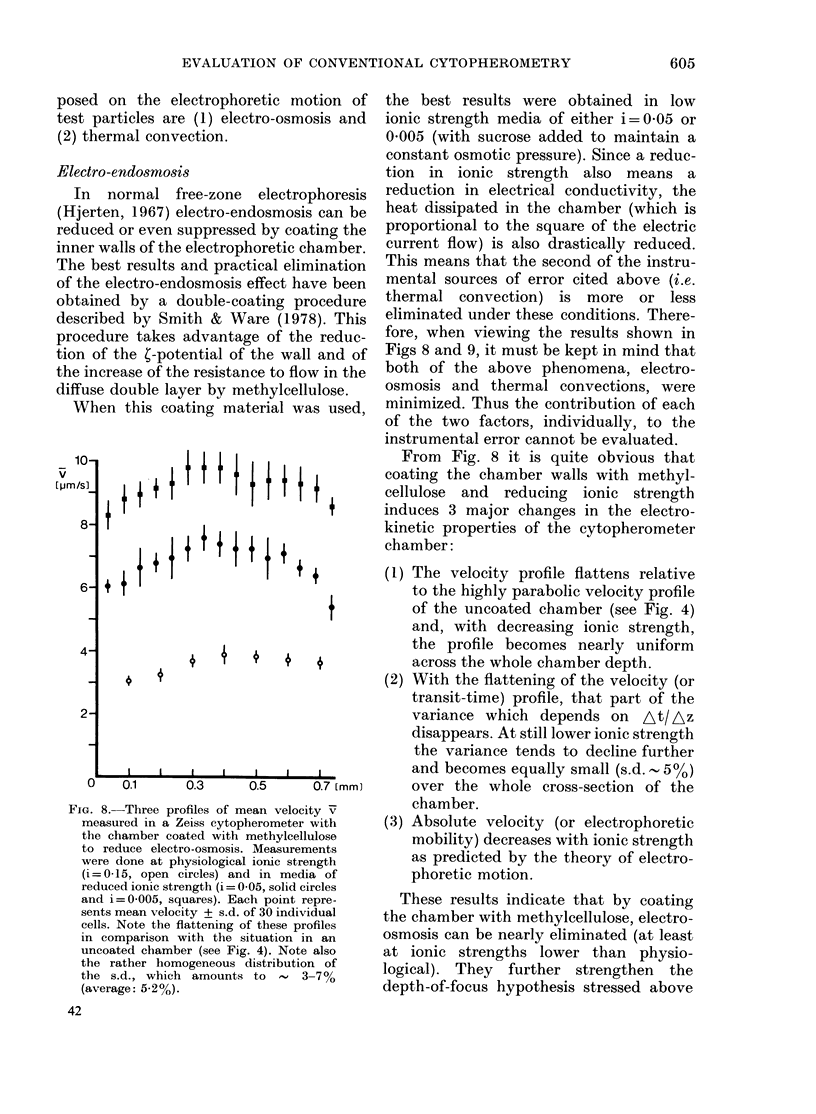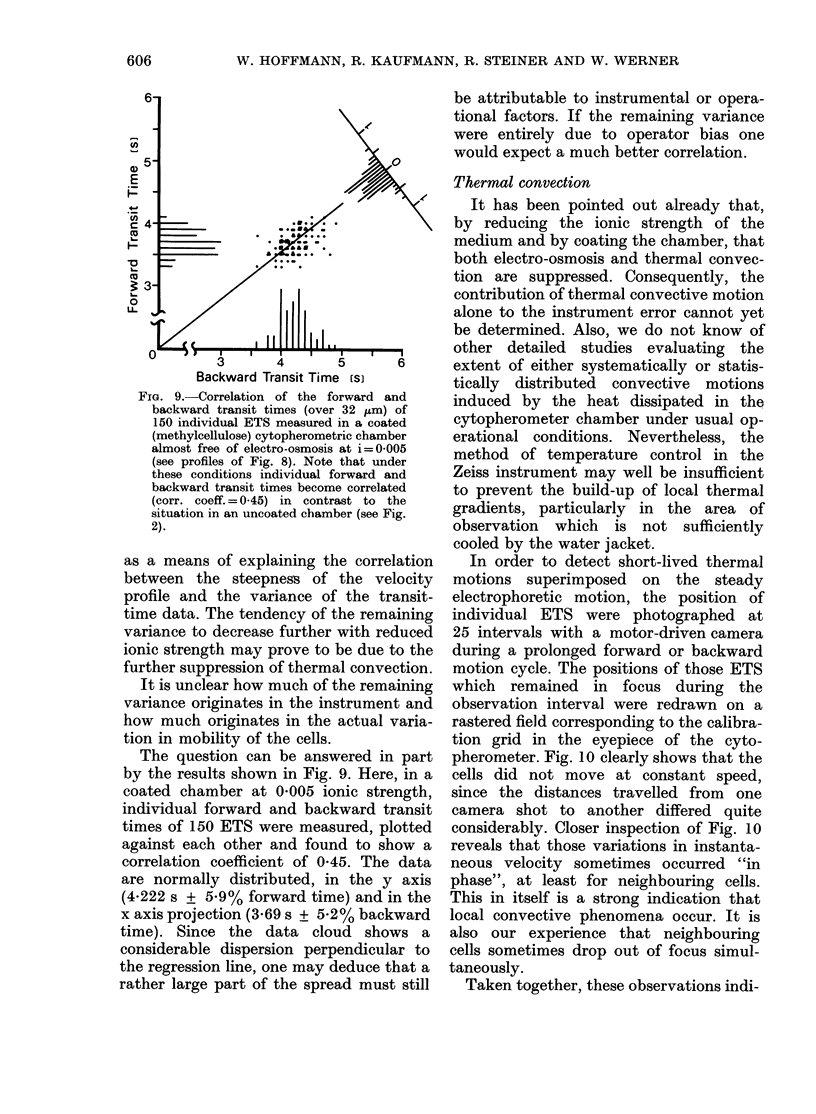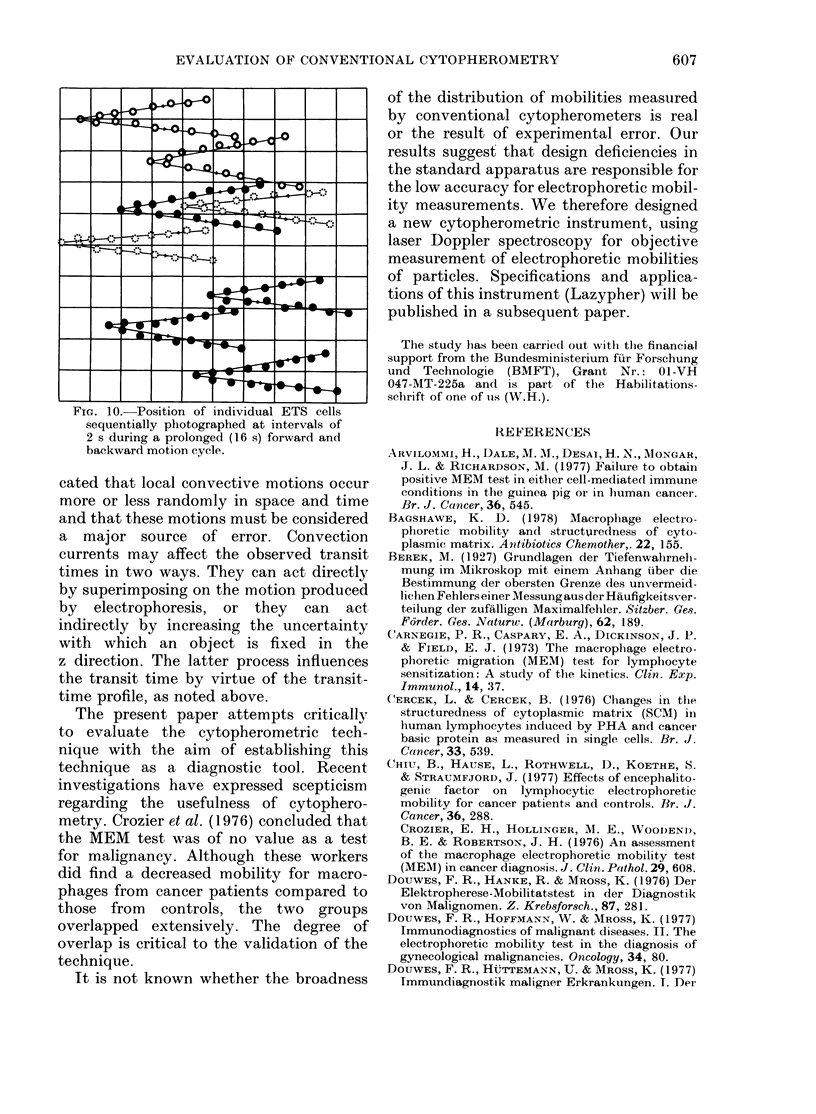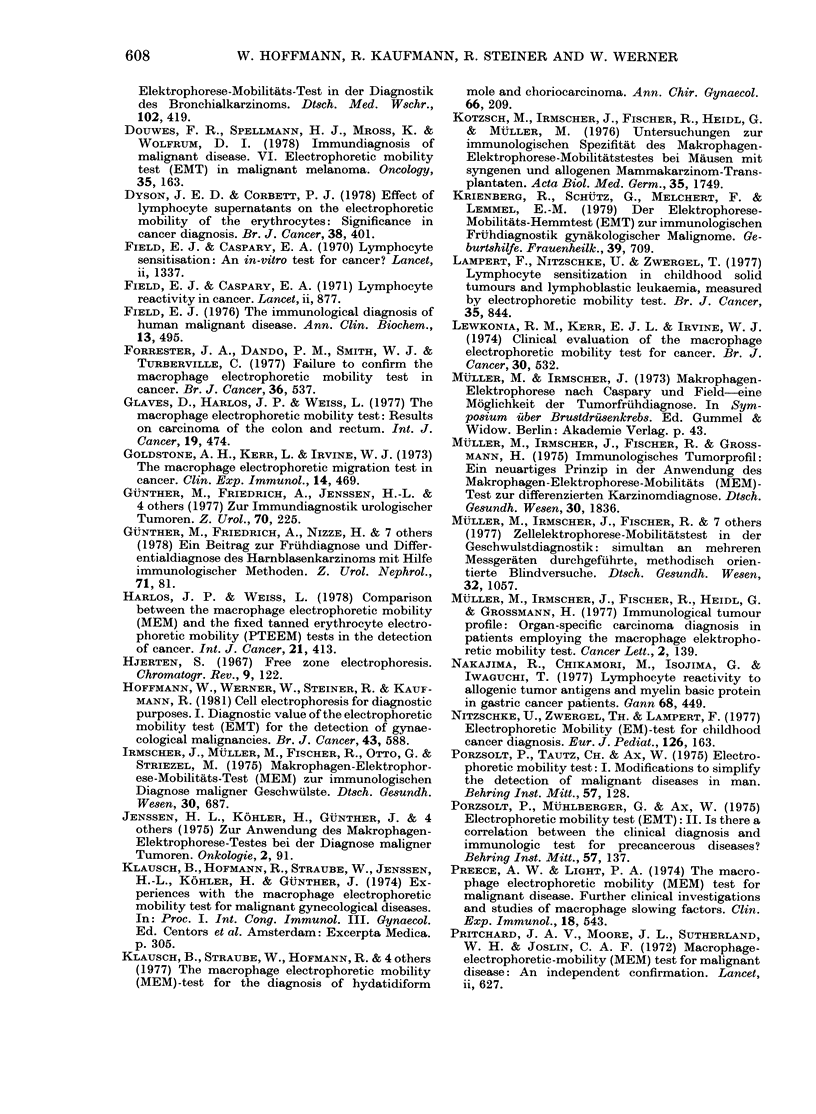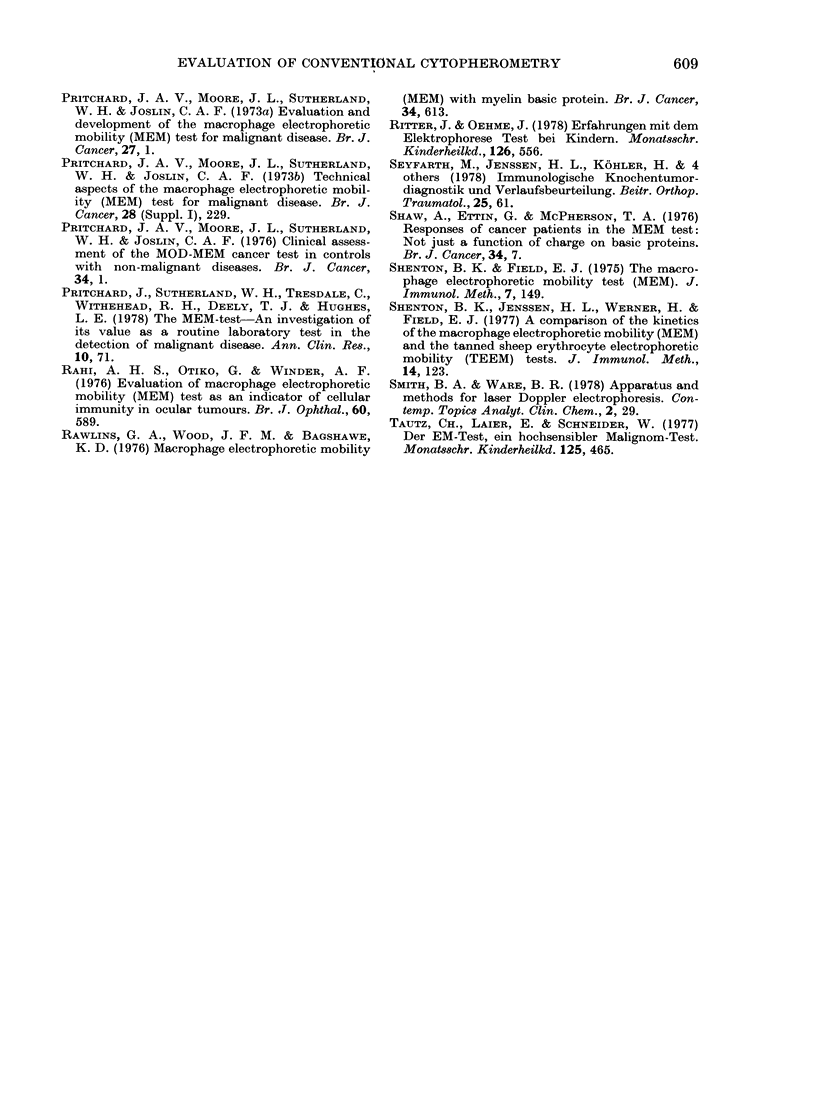# Cell electrophoresis for diagnostic purposes. II. Critical evaluation of conventional cytopherometry.

**DOI:** 10.1038/bjc.1981.89

**Published:** 1981-05

**Authors:** W. Hoffmann, R. Kaufmann, R. Steiner, W. Werner

## Abstract

Determination of the electrophoretic mobility of test cells has been widely used in an attempt to detect so-called lymphokines in a laboratory test for cancer, but operational difficulties are inherent in conventional cytopherometers. This study therefore investigates the technical and operational aspects of cell electrophoresis, using the Zeiss cytopherometer; e.g. influence of electro-osmosis, focus uncertainty, movement due to convection and other sources of error. Implications and possible improvements in the test are discussed.


*Br. J. Cancer* (1981) **43**, 598

### CELL ELECTROPHORESIS FOR DIAGNOSTIC PURPOSES. II. CRITICAL EVALUATION OF CONVENTIONAL CYTOPHEROMETRY

W. HOFFMANN*, R. KAUFMANN†, R. STEINER† AND W. WERNER*

*From the *Frauen- und Poliklinik der Universität Göttingen, D-3400 Göttingen, and the †Physiologisches Institut, Lehrstuhl für Klinische Physiologie der Universität, D-4000 Düsseldorf*

Received 9 January 1981 Accepted 9 February 1981

**Summary.—Determination of the electrophoretic mobility of test cells has been widely used in an attempt to detect so-called lymphokines in a laboratory test for cancer, but operational difficulties are inherent in conventional cytopherometers. This study therefore investigates the technical and operational aspects of cell electrophoresis, using the Zeiss cytopherometer; e.g. influence of electro-osmosis, focus uncertainty, movement due to convection and other sources of error. Implications and possible improvements in the test are discussed.**

DURING THE LAST DECADE, an increasing search has been made for a routine laboratory test for cancer. Recent work in cancer immunology has renewed optimism and led to a proliferation of tests. Most of them depend on the existence of a common immunological denominator: lymphocytes of cancer patients are reported to be "sensitized" and are considered to "react specifically" upon incubation *in vitro* with substances such as basic proteins from brain or carcinoma tissues. Tests have been based either on morphological criteria such as blast-like transformation of the lymphocytes and changes in the structuredness of the protoplasm (Cercek & Cercek, 1976; Bagshawe, 1978) or on the attempt to detect the formation of specific products, usually denoted as lymphokines (see Hoffmann *et al.*, 1981).

Since 1974, with the adaptation of more rigorous test procedures, unsuccessful or conflicting results have been published (see Table).

Any progress towards more reliable and accurate test systems mainly depends on the instrumentation for exact measurement of electrophoretic mobility. In the present paper we therefore reconsider the technical and operational problems and limitations of conventional cytopherometers, using the Zeiss instrument (the instrument most commonly used) as an example.

### MATERIALS AND METHODS

*The cytopherometer set-up.*—The conventional Zeiss cytopherometer set-up was used. It consisted of a microscope stage and an assembly for cell electrophoresis. The optical axis of the microscope is in the horizontal plane. The binocular tube is equipped with 2 eye-pieces (×12·5) and contains an adjustable grid-plate with line distances of 1 mm, so that at the given magnification of ×800 (a tubus factor of 1·6 induced) the travelling distance measured between 2 lines corresponds to 16 μm in the focal plane. The objective lens (Ph ×40/0·65) is suitable for transillumination as well as for phase contrast. Its front lens is attached to the front wall of the temperature-controlled electrophoretic chamber.

A beam divider allowed the connection of a Philips video camera LDH 25/04 and a TV scope. A Wang computer 720C was used for semiautomatic data collection and preprocessing. The time-setting switch simultaneously triggered the electronic stop watch and the reversal of electrode polarity.

*The electrophoretic chamber.*—The chamber was made from K5 optical glass. Dimensions of the rectangular electrophoretic compart-

TABLE I.—*Compilation of results obtained in clinical studies with electrophoretic motility tests (MEM, MOD-MEM, EMT) for the purpose of cancer diagnosis*

| Group* | | | Test procedure | Cytopherometer | Selection of data† | +ve test criteria ‡ | Confirmation§ |
|---|---|---|---|---|---|---|---|
| Newcastle | 1970–77 | Field | MEM, MOD-MEM, EMT | Zeiss | + | −3 to −10 | + |
| Cardiff | 1972–76 | Pritchard | MEM, MOD-MEM | Zeiss | + | −3·5 to −13 | + |
| | 1978 | | MOD-MEM | Rank | + | −10 | − |
| Dresden | 1973–77 | Müller | MOD-MEM | Zeiss | + | −5 to −6 | + |
| Rostock | 1974–78 | Jennsen | MOD-MEM, EMT | Zeiss | | −5 to −10 | + |
| Edinburgh | 1973 | Irvine | MOD-MEM | Zeiss | | −10 | + |
| | 1974 | | MEM, MOD-MEM | Zeiss | + | −5 | − |
| Bristol | 1974 | Preece | MEM | Rank | | −4 | + |
| Marburg | 1975–78 | Ax | EMT | Zeiss | | ±0 to 10 | + |
| Göttingen | 1976–78 | Douwes | EMT | Zeiss | | −5 | + |
| London I | 1976 | Forrester | MOD-MEM | Zeiss | + | | − |
| Belfast | 1976 | Crozier | MOD-MEM | Zeiss | + | −5 | − |
| London II | 1976 | Arvilommi | MOD-MEM | Zeiss | + | −12 | − |
| Edmonton | 1976 | McPherson | MOD-MEM | Rank | + | −5 | + |
| London III | 1976 | Rahi | MOD-MEM | Zeiss | | *t* test | − |
| Tokyo | 1976/77 | Nakajima | MOD-MEM | Sugiura | + | −5 | − |
| Buffalo | 1977, 78 | Weiss | MOD-MEM, EMT | Seaman | + | −5 or *t* test | − |
| London IV | 1977 | Bagshawe | MEM, MOD-MEM | Zeiss | + | −5 | (+) |
| Wisconsin | 1977 | Chiu | MOD-MEM | Zeiss | | ±0 | − |
| Braunschweig | 1978 | Oehme | EMT | Zeiss | | −5 | − |
| Leeds | 1978 | Dyson | EMT | Zeiss mod. | + | −15 | − |
| Mainz | 1979 | Lemmel | EMT | Zeiss | − | −5 | + |

* A "group" is indicated by one author and the assumed permanent location of most of the collaborating authors. For citations see reference list.

† Data were called selected if any kind of rejection procedure was applied to the electrophoretic raw data.

‡ Minimal inhibition of electrophoretic mobility as % of control value, which was taken as a positive test (for cancer).

§ + means that the authors interpret their results as confirming the original paper of Field & Caspary (7).

ment were 35 mm in length, 14 mm in height and 0·7 ± 0·1 mm in depth. On both sides electrode compartments and filling devices were attached with tubes. The inner spaces of the electrode compartments and the electrophoretic compartment were separated by glass sinters covered with a filtering membrane (Sartorius Type 11536). The chamber was filled by bypassing the electrode compartment. The total capacity was ~3·2 ml.

To avoid introduction of air bubbles, the chamber was placed in the vertical position during filling. After the filling, the chamber was returned to the horizontal position and carefully checked for irregularities such as air bubbles and leaks. Filling was considered satisfactory if cells did not move before current was applied. Sedimentation of the cells during electrophoresis was not allowed to exceed half of a grid unit (8 $\mu$m) per grid unit of horizontal travelling distance. Cells exceeding this limit were rejected. If a large portion (>30%) did not fulfil the above criteria the test was stopped and the instrument was refilled.

Unless otherwise stated, electrode compartments were filled with a phosphate-buffered 0·9% NaCl solution, pH 7·3. They were refilled after each assay.

After an overnight break, 3 test runs with test particles (ETS, see below) were made before the instrument was considered ready for use. If the difference ($\triangle$t) between the mean electrophoretic transit times in the forward and the backward directions exceeded 10%, it was assumed that the instrument had failed and the electrophoretic system was completely reassembled and readjusted.

*Solutions used.*—Three different buffer solutions were used: Dulbecco phosphate buffer supplied by Flow Laboratories, and 2 phosphate-buffer solutions at reduced ionic strength (i = 0·05 and 0·005 respectively) prepared in our laboratory. Osmotic pressure was kept constant by the addition of an appropriate amount of sucrose; the pH in each case was adjusted to 7·3 ± 0·1. The solutions were sterilized and stored in sealed bottles until used. Bacteriological checks were carried out routinely.

*Electrophoretic current supply.*—A constant current supply (Zeiss) was applied to the 2 solid platinum electrodes of the electrophoretic assembly. Within the output voltage range of 65–400 V, 2 ranges of current (1–10 mA and 1–50 mA) could be chosen in which the current was kept constant at ±2%. Polarity could be reversed by a manual switch.

*Electrophoretic transit-time measurement.*—Transit times were measured either by a stop watch or, semiautomatically, by push-buttoning the Wang computer. Transit time was defined as the time taken by a cell, travelling in the focal plane of the miscroscope, to pass the distance (16 μm) of two vertical lines of the calibration grid. Accurate measurements of electrophoretic mobility can only be performed with particles moving in one of the so-called stationary layers. Here, according to theory, electro-osmotic flow of the medium is zero and, therefore, should not contribute to the measured motion of the particles. The plane of the anterior stationary layer was determined according to the formula $t \times a$, with

$$a = 0{\cdot}5 - \sqrt{\frac{1}{12} + \frac{32}{K\pi^5}},$$

K denoting the quotient h/t of chamber height (h) and chamber depth (t).

Since the front lens of the objective was attached to the chamber wall, magnification increased when the focus was shifted from the front wall to the rear wall of the chamber. Consequently, the apparent velocity of particle motion increased when focussing beyond the anterior stationary layer. Thus a correction had to be introduced when velocities were measured outside the anterior stationary layer.

FIG. 1.—Histogram of electrophoretic transit times of 500 ETS (lyophilized tanned and salicylated sheep erythrocytes) measured in a Zeiss cytopherometer.

*ETS-sheep erythrocytes.*—ETS indicator cells (tanned and salicylated erythrocytes from sheep) were purchased as a dry preparation commercially available from Behring Werke, Marburg.

The lyophilized erythrocytes were reconstituted with 1 ml of distilled water, and then prepared for use according to the manufacturer's directions.

### RESULTS AND DISCUSSION

### *Accuracy limits of electrophoretic mobility data measured in the Zeiss cytopherometer*

The following measurements were made under experimental conditions optimized to the best of our knowledge and skill. All potential sources of drift (air bubbles, cracks, defective seals) were carefully eliminated, current flow was monitored and checked to be the same at both polarities, and temperature control was optimized as far as possible under the geometric conditions given.

The electrophoretic transit times of 500 ETS cells measured in the Zeiss cytopherometer under "optimal conditions", showed a normal distribution (see Fig. 1) around a mean transit time of 3·862 s. Standard deviation was 0·365 or ± 9·1%. In the absence of any cross-check data, it was difficult to decide whether this transit-time distribution represented the true mobility variation of the investigated ETS cells or the statistical spread inherent to the instrument and/or the measuring procedure itself. However, one might obtain indirect evidence by checking whether or not the forward and backward transit times of individual cells are correlated. Electrophoretic transit times of 200 individual ETS were therefore measured in three different layers of the cytopherometric chamber (Fig.).

The forward time of each cell was plotted against its backward time. One does not need a computer to see from the 3 data clouds that there is no correlation between forward and backward times.

Fig. 2.—Date clouds of individual transit times of 200 ETS each. Measurement done by experienced investigator in the nominally stationary layer (b) and in a layer 100 $\mu$m in front of (a) or behind (c) the stationary layer.

Fig. 3.—Significance borders for electrophoretic mobility tests in a Zeiss cytopherometer based on a Gaussian distribution of data, as in Fig. 1. The curves indicate the minimum inhibition of mean transit times (as a percentage of control value) which can be measured with a given number of individual test cells.

The same result was obtained in 11 out of 12 sets of identical measurements (involving 3 different investigators). In only one case was a correlation coefficient > 0·20 (0·30) between forward and backward transit times obtained.

This result strongly indicates that the mobility spread shown in Fig. 1 must be attributed to one or several operational or instrumental factors which affected the true electrophoretic mobility, obviously at random, but to such an extent that the true variance of cell mobility was completely lost in the much broader variances imposed by the instrument or the operator. We will consider this point later.

If, for the moment, one accepts the above variance as valid, one may ask what differences in mean transit times ($\Delta\bar{t}$) can be determined with a statistically acceptable error probability of say $P < 0{\cdot}01$ or $< 0{\cdot}05$. The answer can be easily deduced from Fig. 3.

With the usual number of particles measured (N = 15) the limit of $\Delta\bar{t}$ which can be determined with $P < 0{\cdot}05$ is $\Delta\bar{t} \sim 7\%$; for an error probability of $P < 0{\cdot}01$ this limit increases to $\Delta\bar{t} \sim 10\%$. Both figures are at or above the value of the slowing effect many investigators consider to be decisive for a positive test.

One may argue that accuracy increases with an increasing number of particles measured. However, since this is a square-root law (accuracy is proportional to $\sqrt{N}$), considerably better discrimination would require increasing N by a factor of 5–10. However, such an increase in measurements would make the test even more time-consuming than it already is.

*Operational sources of error (timing, depth of focus uncertainty, inter-assay accuracy)*

In the following we define a source of error as "operational" if it involves the usual perception or physical reaction of the investigator; it is called "instrumental" if it is based on physical principles affecting or disturbing the electrophoretic motion of the particles measured.

*Timing*

One obvious operational source of error might be the inaccuracy of timing (done either traditionally or by push-buttoning a semiautomatic device). If we assume

FIG. 4.—Profile of mean transit time $\bar{t}$ (dashed line) and electrophoretic velocity $\bar{v}$ (solid line) for ETS obtained in the Zeiss cytopherometer under normal instrumental conditions (see Methods). Each point represents mean $\pm$s.d. of 30 individual cells. s.l. = stationary layer.

that an experienced investigator will stay within $\pm 0{\cdot}1$ s absolute error limits, the resulting s.d. of $\sim 2\%$ is clearly not sufficient to explain the variance shown in Fig. 1.

The argument is further ruled out by the results shown in Fig. 4, which shows the transit time and velocity profile of ETS measured across the whole depth of the cytopherometer chamber. As expected these are parabolic or quasiparabolic profiles with their shortest transit times (or highest velocities) in the middle of the chamber and exponentially rising transit times toward both front and back chamber walls. These profiles are well known, and can be explained on the basis of two electro-osmotic circular movements of the medium slowing (or even inverting) the electrophoretic motion of the test particles close to the chamber walls, and accelerating them in the middle part of the chamber. It is interesting to note in this figure that the value of s.d. increased systematically and drastically as the plane of measurement approached either wall. This increase was much higher than one would expect from the increase in the absolute values.

If inaccuracy of timing were indeed a major factor, the mobility spread should not depend on chamber depth in such a clear-cut and obvious fashion.

*Depth of focus uncertainty*

It is proposed that the results shown in Fig. 4 are due to another source of error which is basically operational in nature. Although all the particles may appear to the operator to lie in the same plane (the plane of optical focus) they may, in fact, be moving in slightly different planes or may change planes while traversing the chamber. In either case, the measured transit time will be influenced by the steepness of the transit-time profile ($\Delta t/\Delta z$).

In cytopherometric studies it is tacitly assumed that the virtual depth of focus corresponds to the nominal depth of focus, basically given by the numeral aperture (A) of the objective and the useful magnification ($\beta$). For the $\times$ 40/0·65 objective used in the standard version of the Zeiss cytopherometer, a nominal focus depth or axial resolution of 2·5 $\mu$m can be derived at $\beta = 800$ (Berek, 1927). Accommodation by the operator's eye may enlarge that nominal depth of focus. Thus a human component must be added to the nominal depth of focus, which, depending on the individual's visual status, may be estimated as 0·5–2 $\mu$m. The composite depth of field is 3·0–4·5 $\mu$m. This value is only valid when the object under observation possesses an optimally contrasting structure with dimensions close to the lateral resolution of the microscope. In the case of the cytopherometer loaded with ETS (or macrophages) the lateral resolution is about 0.6 $\mu$m (at $\lambda = 550$ nm) but an optimally contrasting structuredness of that dimension is missing in the cytoplasm of both kinds of particles.

One must also take into account that the diameter of the test particles (ETS or macrophages) exceeds the above depth of focus by a factor of 2–3. Since the cell borders are obviously the only optimally contrasting and laterally resolved structures, one ends up with the situation illustrated in Fig. 5. From this an operational focus depth of 10–12 $\mu$m may be deduced. This means that cells may change their axial position within these limits before the investigator realizes that they have dropped out of focus.

Since the steepness of the transit-time profile at the intersection with the stationary layer, $\Delta t/\Delta z$, $\sim$0·03 s/$\mu$m (*i.e.* roughly a 1% transit-time change per $\mu$m drift in the z direction), the error introduced by this depth of focus uncertainty may reach $\pm$6%. One must bear in mind,

FIG. 5.—Schematic drawing illustrating the problem of "focal depth uncertainty" in the cytopherometer. The nominal focal depth (n) which is given by the numerical aperture of the objective lens and the useful magnification, is broadened by a "physiological component" given by the accommodation limits of the operator's eye. Thus a composite focal depth (c) is defined. Since the diameter of (the spherical) ETS (2r $\sim$ 7–8 $\mu$m) exceeds the limits of ($\sim$ 3–4·5 $\mu$m) their axial position may vary over a *virtual* focal depth (v) of $\simeq$10–12 $\mu$m before the operator's eye will register an "out of focus" situation. The two circles indicate the extreme anterior and posterior position where the cell extends far enough into the layer c to stay in focus with either its circumference $S_2$ or $S_1$, the radius of which differs from the spherical radius by a distance (d) equal to the lateral resolution of the optical system.

FIG. 6.—Correlation between the steepness of the electrophoretic transit-time profile ($\Delta t/\Delta z$) and the variance ($\tau$) of the mean transit time ($\bar{t}$) measured in the Zeiss cytopherometer under normal instrumental and operational conditions (see Methods). Data were taken from 5 mean transit-time profiles as shown in Fig. 4. Note that with $\Delta t/\Delta z$ approaching zero (*i.e.* under conditions where t becomes independent from z as, for instance, in the middle of the chamber), the regression line intersects the y axis at a $\tau$ of $\sim$ 5% which appears to be the s.d. remaining after elimination of electro-osmosis (see Fig. 8).

however, that the subjective criteria for finding a microscopic object outside or inside a focus plane (which, in reality, is a layer) may inject additional operator-induced variances into the cytopherometer data.

The argument that part of the variance must indeed be related to the expansion of the z dimension of the subjective focus layer beyond the limits given by classical optics is, at least inferentially, indicated by the obvious correlation between the variance and the steepness of the transit-time profile (see Fig. 6). In the following section we will show transit-time profiles measured in cytopherometer chambers almost free of electro-osmosis and, hence, with an almost homogeneous velocity profile across the whole chamber depth (see Fig. 8). Here, where $\triangle t/\triangle z$ approaches zero, and transit times become independent of focal plane, variances are small and, more importantly, become independent of chamber depth.

Recently, Schmoll (private communication) has attempted to "sharpen" the out-of-focus criterion by employing a TV system which, by electronic processing of the video signal, can detect any drift of particles beyond the nominal focal depth of 2·5 $\mu$m. It was found that this system rejected two-thirds of the cells which would have been accepted by an experienced investigator. At the same time, the s.d. of the remaining one-third (truly "stationary") cells dropped from initially 9–10% (when applying a selective focus criterion) to 5% (N = 50), which is about the same figure as determined in the present investigation (see Fig. 6).

*Day-to-Day variability*

It is common experience that an acceptable reproducibility of cytopherometric data is difficult to establish. Some investigators claim that they have reached a variance in their control data of a few *per cent* (Tautz, private communication); others, however, are unable to bring their systems to satisfactory long-term stability.

An example of what we achieved after optimizing all operational and instrumental conditions is shown in Fig. 7. It shows the data obtained in 14 successive (and not selected) measurements of ETS mean transit times performed by an experienced investigator. After an over-night break (indicated in the diagram) the cytopherometer chamber was disassembled, cleaned, refilled and readjusted in the cytopherometer. The overall variability (between assays as well as from day to day) derived from 3 sequences of such measurements produced an s.d. of 5%. The influence of operator idiosyncrasies on transit-time measurements was not explored, since this was not deemed germane to the present investigation, nor important enough to justify the large amount of time and effort needed for this kind of study.

FIG. 7.—Variation of mean transit time ($\bar{t}$) in S and the difference in mean transit forward and backward time ($\triangle\bar{t}$) obtained in 14 successive measurements with ETS in a Zeiss cytopherometer under normal instrumental and operational conditions (see Methods). Each point of ($\bar{t}$) represents the mean of forward and backward times of 30 individual cells. Arrows indicate overnight breaks or new measurement (NE) on the same day.

*Instrumental sources of error*

As mentioned above, in a properly operated cytopherometer the remaining sources of kinetic phenomena superimposed on the electrophoretic motion of test particles are (1) electro-osmosis and (2) thermal convection.

*Electro-endosmosis*

In normal free-zone electrophoresis (Hjerten, 1967) electro-endosmosis can be reduced or even suppressed by coating the inner walls of the electrophoretic chamber. The best results and practical elimination of the electro-endosmosis effect have been obtained by a double-coating procedure described by Smith & Ware (1978). This procedure takes advantage of the reduction of the $\zeta$-potential of the wall and of the increase of the resistance to flow in the diffuse double layer by methylcellulose.

When this coating material was used,

FIG. 8.—Three profiles of mean velocity $\bar{v}$ measured in a Zeiss cytopherometer with the chamber coated with methylcellulose to reduce electro-osmosis. Measurements were done at physiological ionic strength (i = 0·15, open circles) and in media of reduced ionic strength (i = 0·05, solid circles and i = 0·005, squares). Each point represents mean velocity ± s.d. of 30 individual cells. Note the flattening of these profiles in comparison with the situation in an uncoated chamber (see Fig. 4). Note also the rather homogeneous distribution of the s.d., which amounts to ~ 3–7% (average: 5·2%).

the best results were obtained in low ionic strength media of either i = 0·05 or 0·005 (with sucrose added to maintain a constant osmotic pressure). Since a reduction in ionic strength also means a reduction in electrical conductivity, the heat dissipated in the chamber (which is proportional to the square of the electric current flow) is also drastically reduced. This means that the second of the instrumental sources of error cited above (*i.e.* thermal convection) is more or less eliminated under these conditions. Therefore, when viewing the results shown in Figs 8 and 9, it must be kept in mind that both of the above phenomena, electro-osmosis and thermal convections, were minimized. Thus the contribution of each of the two factors, individually, to the instrumental error cannot be evaluated.

From Fig. 8 it is quite obvious that coating the chamber walls with methylcellulose and reducing ionic strength induces 3 major changes in the electrokinetic properties of the cytopherometer chamber:

(1) The velocity profile flattens relative to the highly parabolic velocity profile of the uncoated chamber (see Fig. 4) and, with decreasing ionic strength, the profile becomes nearly uniform across the whole chamber depth.
(2) With the flattening of the velocity (or transit-time) profile, that part of the variance which depends on $\triangle t/\triangle z$ disappears. At still lower ionic strength the variance tends to decline further and becomes equally small (s.d. ~ 5%) over the whole cross-section of the chamber.
(3) Absolute velocity (or electrophoretic mobility) decreases with ionic strength as predicted by the theory of electrophoretic motion.

These results indicate that by coating the chamber with methylcellulose, electro-osmosis can be nearly eliminated (at least at ionic strengths lower than physiological). They further strengthen the depth-of-focus hypothesis stressed above

FIG. 9.—Correlation of the forward and backward transit times (over 32 μm) of 150 individual ETS measured in a coated (methylcellulose) cytopherometric chamber almost free of electro-osmosis at i = 0·005 (see profiles of Fig. 8). Note that under these conditions individual forward and backward transit times become correlated (corr. coeff. = 0·45) in contrast to the situation in an uncoated chamber (see Fig. 2).

as a means of explaining the correlation between the steepness of the velocity profile and the variance of the transit-time data. The tendency of the remaining variance to decrease further with reduced ionic strength may prove to be due to the further suppression of thermal convection.

It is unclear how much of the remaining variance originates in the instrument and how much originates in the actual variation in mobility of the cells.

The question can be answered in part by the results shown in Fig. 9. Here, in a coated chamber at 0·005 ionic strength, individual forward and backward transit times of 150 ETS were measured, plotted against each other and found to show a correlation coefficient of 0·45. The data are normally distributed, in the y axis (4·222 s ± 5·9% forward time) and in the x axis projection (3·69 s ± 5·2% backward time). Since the data cloud shows a considerable dispersion perpendicular to the regression line, one may deduce that a rather large part of the spread must still be attributable to instrumental or operational factors. If the remaining variance were entirely due to operator bias one would expect a much better correlation.

*Thermal convection*

It has been pointed out already that, by reducing the ionic strength of the medium and by coating the chamber, that both electro-osmosis and thermal convection are suppressed. Consequently, the contribution of thermal convective motion alone to the instrument error cannot yet be determined. Also, we do not know of other detailed studies evaluating the extent of either systematically or statistically distributed convective motions induced by the heat dissipated in the cytopherometer chamber under usual operational conditions. Nevertheless, the method of temperature control in the Zeiss instrument may well be insufficient to prevent the build-up of local thermal gradients, particularly in the area of observation which is not sufficiently cooled by the water jacket.

In order to detect short-lived thermal motions superimposed on the steady electrophoretic motion, the position of individual ETS were photographed at 25 intervals with a motor-driven camera during a prolonged forward or backward motion cycle. The positions of those ETS which remained in focus during the observation interval were redrawn on a rastered field corresponding to the calibration grid in the eyepiece of the cytopherometer. Fig. 10 clearly shows that the cells did not move at constant speed, since the distances travelled from one camera shot to another differed quite considerably. Closer inspection of Fig. 10 reveals that those variations in instantaneous velocity sometimes occurred "in phase", at least for neighbouring cells. This in itself is a strong indication that local convective phenomena occur. It is also our experience that neighbouring cells sometimes drop out of focus simultaneously.

Taken together, these observations indi-

FIG. 10.—Position of individual ETS cells sequentially photographed at intervals of 2 s during a prolonged (16 s) forward and backward motion cycle.

cated that local convective motions occur more or less randomly in space and time and that these motions must be considered a major source of error. Convection currents may affect the observed transit times in two ways. They can act directly by superimposing on the motion produced by electrophoresis, or they can act indirectly by increasing the uncertainty with which an object is fixed in the z direction. The latter process influences the transit time by virtue of the transit-time profile, as noted above.

The present paper attempts critically to evaluate the cytopherometric technique with the aim of establishing this technique as a diagnostic tool. Recent investigations have expressed scepticism regarding the usefulness of cytopherometry. Crozier *et al.* (1976) concluded that the MEM test was of no value as a test for malignancy. Although these workers did find a decreased mobility for macrophages from cancer patients compared to those from controls, the two groups overlapped extensively. The degree of overlap is critical to the validation of the technique.

It is not known whether the broadness of the distribution of mobilities measured by conventional cytopherometers is real or the result of experimental error. Our results suggest that design deficiencies in the standard apparatus are responsible for the low accuracy for electrophoretic mobility measurements. We therefore designed a new cytopherometric instrument, using laser Doppler spectroscopy for objective measurement of electrophoretic mobilities of particles. Specifications and applications of this instrument (Lazypher) will be published in a subsequent paper.

The study has been carried out with the financial support from the Bundesministerium für Forschung und Technologie (BMFT), Grant Nr.: 01-VH 047-MT-225a and is part of the Habilitationsschrift of one of us (W.H.).

### REFERENCES

ARVILOMMI, H., DALE, M. M., DESAI, H. N., MONGAR, J. L. & RICHARDSON, M. (1977) Failure to obtain positive MEM test in either cell-mediated immune conditions in the guinea pig or in human cancer. *Br. J. Cancer*, **36**, 545.

BAGSHAWE, K. D. (1978) Macrophage electrophoretic mobility and structuredness of cytoplasmic matrix. *Antibiotics Chemother,*. **22**, 155.

BEREK, M. (1927) Grundlagen der Tiefenwahrnehmung im Mikroskop mit einem Anhang über die Bestimmung der obersten Grenze des unvermeidlichen Fehlers einer Messung aus der Häufigkeitsverteilung der zufälligen Maximalfehler. *Sitzber. Ges. Förder. Ges. Naturw.* (*Marburg*), **62**, 189.

CARNEGIE, P. R., CASPARY, E. A., DICKINSON, J. P. & FIELD, E. J. (1973) The macrophage electrophoretic migration (MEM) test for lymphocyte sensitization: A study of the kinetics. *Clin. Exp. Immunol.*, **14**, 37.

CERCEK, L. & CERCEK, B. (1976) Changes in the structuredness of cytoplasmic matrix (SCM) in human lymphocytes induced by PHA and cancer basic protein as measured in single cells. *Br. J. Cancer*, **33**, 539.

CHIU, B., HAUSE, L., ROTHWELL, D., KOETHE, S. & STRAUMFJORD, J. (1977) Effects of encephalitogenic factor on lymphocytic electrophoretic mobility for cancer patients and controls. *Br. J. Cancer*, **36**, 288.

CROZIER, E. H., HOLLINGER, M. E., WOODEND, B. E. & ROBERTSON, J. H. (1976) An assessment of the macrophage electrophoretic mobility test (MEM) in cancer diagnosis. *J. Clin. Pathol.* **29**, 608.

DOUWES, F. R., HANKE, R. & MROSS, K. (1976) Der Elektropherese-Mobilitatstest in der Diagnostik von Malignomen. *Z. Krebsforsch.*, **87**, 281.

DOUWES, F. R., HOFFMANN, W. & MROSS, K. (1977) Immunodiagnostics of malignant diseases. II. The electrophoretic mobility test in the diagnosis of gynecological malignancies. *Oncology*, **34**, 80.

DOUWES, F. R., HÜTTEMANN, U. & MROSS, K. (1977) Immundiagnostik maligner Erkrankungen. I. Der

Elektrophorese-Mobilitäts-Test in der Diagnostik des Bronchialkarzinoms. *Dtsch. Med. Wschr.*, **102**, 419.

DOUWES, F. R., SPELLMANN, H. J., MROSS, K. & WOLFRUM, D. I. (1978) Immundiagnosis of malignant disease. VI. Electrophoretic mobility test (EMT) in malignant melanoma. *Oncology*, **35**, 163.

DYSON, J. E. D. & CORBETT, P. J. (1978) Effect of lymphocyte supernatants on the electrophoretic mobility of the erythrocytes: Significance in cancer diagnosis. *Br. J. Cancer*, **38**, 401.

FIELD, E. J. & CASPARY, E. A. (1970) Lymphocyte sensitisation: An *in-vitro* test for cancer? *Lancet*, ii, 1337.

FIELD, E. J. & CASPARY, E. A. (1971) Lymphocyte reactivity in cancer. *Lancet*, ii, 877.

FIELD, E. J. (1976) The immunological diagnosis of human malignant disease. *Ann. Clin. Biochem.*, **13**, 495.

FORRESTER, J. A., DANDO, P. M., SMITH, W. J. & TURBERVILLE, C. (1977) Failure to confirm the macrophage electrophoretic mobility test in cancer. *Br. J. Cancer*, **36**, 537.

GLAVES, D., HARLOS, J. P. & WEISS, L. (1977) The macrophage electrophoretic mobility test: Results on carcinoma of the colon and rectum. *Int. J. Cancer*, **19**, 474.

GOLDSTONE, A. H., KERR, L. & IRVINE, W. J. (1973) The macrophage electrophoretic migration test in cancer. *Clin. Exp. Immunol.*, **14**, 469.

GÜNTHER, M., FRIEDRICH, A., JENSSEN, H.-L. & 4 others (1977) Zur Immundiagnostik urologischer Tumoren. *Z. Urol.*, **70**, 225.

GÜNTHER, M., FRIEDRICH, A., NIZZE, H. & 7 others (1978) Ein Beitrag zur Frühdiagnose und Differentialdiagnose des Harnblasenkarzinoms mit Hilfe immunologischer Methoden. *Z. Urol. Nephrol.*, **71**, 81.

HARLOS, J. P. & WEISS, L. (1978) Comparison between the macrophage electrophoretic mobility (MEM) and the fixed tanned erythrocyte electrophoretic mobility (PTEEM) tests in the detection of cancer. *Int. J. Cancer*, **21**, 413.

HJERTEN, S. (1967) Free zone electrophoresis. *Chromatogr. Rev.*, **9**, 122.

HOFFMANN, W., WERNER, W., STEINER, R. & KAUFMANN, R. (1981) Cell electrophoresis for diagnostic purposes. I. Diagnostic value of the electrophoretic mobility test (EMT) for the detection of gynaecological malignancies. *Br. J. Cancer*, **43**, 588.

IRMSCHER, J., MÜLLER, M., FISCHER, R., OTTO, G. & STRIEZEL, M. (1975) Makrophagen-Elektrophorese-Mobilitäts-Test (MEM) zur immunologischen Diagnose maligner Geschwülste. *Dtsch. Gesundh. Wesen*, **30**, 687.

JENSSEN, H. L., KÖHLER, H., GÜNTHER, J. & 4 others (1975) Zur Anwendung des Makrophagen-Elektrophorese-Testes bei der Diagnose maligner Tumoren. *Onkologie*, **2**, 91.

KLAUSCH, B., HOFMANN, R., STRAUBE, W., JENSSEN, H.-L., KÖHLER, H. & GÜNTHER, J. (1974) Experiences with the macrophage electrophoretic mobility test for malignant gynecological diseases. In: *Proc. I. Int. Cong. Immunol. III. Gynaecol.* Ed. Centors *et al.* Amsterdam: Excerpta Medica. p. 305.

KLAUSCH, B., STRAUBE, W., HOFMANN, R. & 4 others (1977) The macrophage electrophoretic mobility (MEM)-test for the diagnosis of hydatidiform mole and choriocarcinoma. *Ann. Chir. Gynaecol.* **66**, 209.

KOTZSCH, M., IRMSCHER, J., FISCHER, R., HEIDL, G. & MÜLLER, M. (1976) Untersuchungen zur immunologischen Spezifität des Makrophagen-Elektrophorese-Mobilitätstestes bei Mäusen mit syngenen und allogenen Mammakarzinom-Transplantaten. *Acta Biol. Med. Germ.*, **35**, 1749.

KRIENBERG, R., SCHÜTZ, G., MELCHERT, F. & LEMMEL, E.-M. (1979) Der Elektrophorese-Mobilitäts-Hemmtest (EMT) zur immunologischen Frühdiagnostik gynäkologischer Malignome. *Geburtshilfe. Frauenheilk.*, **39**, 709.

LAMPERT, F., NITZSCHKE, U. & ZWERGEL, T. (1977) Lymphocyte sensitization in childhood solid tumours and lymphoblastic leukaemia, measured by electrophoretic mobility test. *Br. J. Cancer*, **35**, 844.

LEWKONIA, R. M., KERR, E. J. L. & IRVINE, W. J. (1974) Clinical evaluation of the macrophage electrophoretic mobility test for cancer. *Br. J. Cancer*, **30**, 532.

MÜLLER, M. & IRMSCHER, J. (1973) Makrophagen-Elektrophorese nach Caspary und Field—eine Möglichkeit der Tumorfrühdiagnose. In *Symposium über Brustdrüsenkrebs*. Ed. Gummel & Widow. Berlin: Akademie Verlag. p. 43.

MÜLLER, M., IRMSCHER, J., FISCHER, R. & GROSSMANN, H. (1975) Immunologisches Tumorprofil: Ein neuartiges Prinzip in der Anwendung des Makrophagen-Elektrophorese-Mobilitäts (MEM)-Test zur differenzierten Karzinomdiagnose. *Dtsch. Gesundh. Wesen*, **30**, 1836.

MÜLLER, M., IRMSCHER, J., FISCHER, R. & 7 others (1977) Zellelektrophorese-Mobilitätstest in der Geschwulstdiagnostik: simultan an mehreren Messgeräten durchgeführte, methodisch orientierte Blindversuche. *Dtsch. Gesundh. Wesen*, **32**, 1057.

MÜLLER, M., IRMSCHER, J., FISCHER, R., HEIDL, G. & GROSSMANN, H. (1977) Immunological tumour profile: Organ-specific carcinoma diagnosis in patients employing the macrophage elektrophoretic mobility test. *Cancer Lett.*, **2**, 139.

NAKAJIMA, R., CHIKAMORI, M., ISOJIMA, G. & IWAGUCHI, T. (1977) Lymphocyte reactivity to allogenic tumor antigens and myelin basic protein in gastric cancer patients. *Gann* **68**, 449.

NITZSCHKE, U., ZWERGEL, TH. & LAMPERT, F. (1977) Electrophoretic Mobility (EM)-test for childhood cancer diagnosis. *Eur. J. Pediat.*, **126**, 163.

PORZSOLT, P., TAUTZ, CH. & AX, W. (1975) Electrophoretic mobility test: I. Modifications to simplify the detection of malignant diseases in man. *Behring Inst. Mitt.*, **57**, 128.

PORZSOLT, P., MÜHLBERGER, G. & AX, W. (1975) Electrophoretic mobility test (EMT): II. Is there a correlation between the clinical diagnosis and immunologic test for precancerous diseases? *Behring Inst. Mitt.*, **57**, 137.

PREECE, A. W. & LIGHT, P. A. (1974) The macrophage electrophoretic mobility (MEM) test for malignant disease. Further clinical investigations and studies of macrophage slowing factors. *Clin. Exp. Immunol.*, **18**, 543.

PRITCHARD, J. A. V., MOORE, J. L., SUTHERLAND, W. H. & JOSLIN, C. A. F. (1972) Macrophage-electrophoretic-mobility (MEM) test for malignant disease: An independent confirmation. *Lancet*, ii, 627.

PRITCHARD, J. A. V., MOORE, J. L., SUTHERLAND, W. H. & JOSLIN, C. A. F. (1973*a*) Evaluation and development of the macrophage electrophoretic mobility (MEM) test for malignant disease. *Br. J. Cancer*, **27**, 1.

PRITCHARD, J. A. V., MOORE, J. L., SUTHERLAND, W. H. & JOSLIN, C. A. F. (1973*b*) Technical aspects of the macrophage electrophoretic mobility (MEM) test for malignant disease. *Br. J. Cancer*, **28** (Suppl. I), 229.

PRITCHARD, J. A. V., MOORE, J. L., SUTHERLAND, W. H. & JOSLIN, C. A. F. (1976) Clinical assessment of the MOD-MEM cancer test in controls with non-malignant diseases. *Br. J. Cancer*, **34**, 1.

PRITCHARD, J., SUTHERLAND, W. H., TRESDALE, C., WITHEHEAD, R. H., DEELY, T. J. & HUGHES, L. E. (1978) The MEM-test—An investigation of its value as a routine laboratory test in the detection of malignant disease. *Ann. Clin. Res.*, **10**, 71.

RAHI, A. H. S., OTIKO, G. & WINDER, A. F. (1976) Evaluation of macrophage electrophoretic mobility (MEM) test as an indicator of cellular immunity in ocular tumours. *Br. J. Ophthal.*, **60**, 589.

RAWLINS, G. A., WOOD, J. F. M. & BAGSHAWE, K. D. (1976) Macrophage electrophoretic mobility (MEM) with myelin basic protein. *Br. J. Cancer*, **34**, 613.

RITTER, J. & OEHME, J. (1978) Erfahrungen mit dem Elektrophorese Test bei Kindern. *Monatsschr. Kinderheilkd.*, **126**, 556.

SEYFARTH, M., JENSSEN, H. L., KÖHLER, H. & 4 others (1978) Immunologische Knochentumordiagnostik und Verlaufsbeurteilung. *Beitr. Orthop. Traumatol.*, **25**, 61.

SHAW, A., ETTIN, G. & MCPHERSON, T. A. (1976) Responses of cancer patients in the MEM test: Not just a function of charge on basic proteins. *Br. J. Cancer*, **34**, 7.

SHENTON, B. K. & FIELD, E. J. (1975) The macrophage electrophoretic mobility test (MEM). *J. Immunol. Meth.*, **7**, 149.

SHENTON, B. K., JENSSEN, H. L., WERNER, H. & FIELD, E. J. (1977) A comparison of the kinetics of the macrophage electrophoretic mobility (MEM) and the tanned sheep erythrocyte electrophoretic mobility (TEEM) tests. *J. Immunol. Meth.*, **14**, 123.

SMITH, B. A. & WARE, B. R. (1978) Apparatus and methods for laser Doppler electrophoresis. *Contemp. Topics Analyt. Clin. Chem.*, **2**, 29.

TAUTZ, CH., LAIER, E. & SCHNEIDER, W. (1977) Der EM-Test, ein hochsensibler Malignom-Test. *Monatsschr. Kinderheilkd.* **125**, 465.